# High Effective of 14-Day High-Dose PPI- Bismuth-Containing Quadruple Therapy with Probiotics Supplement for *Helicobacter Pylori* Eradication: A Double Blinded-Randomized Placebo-Controlled Study

**DOI:** 10.31557/APJCP.2019.20.9.2859

**Published:** 2019

**Authors:** Piyakorn Poonyam, Peranart Chotivitayatarakorn, Ratha-Korn Vilaichone

**Affiliations:** 1 *Gastroenterology Unit,*; 2 *Digestive Diseases Research Center (DRC), Thammasat University Hospital,*; 3 *Department of Medicine, Chulabhorn International College of Medicine (CICM) at Thammasat University, Pathumthani, Thailand. *

**Keywords:** High-dose PPI, quadruple therapy, probiotics supplement, Helicobacter pylori

## Abstract

**Background:**

*Helicobacter pylori* (*H. pylori*) infection is important risk factors for chronic gastritis, peptic ulcer and gastric cancer. Bismuth-containing quadruple therapy has recently been the first-line regimen recommended in many European countries but has limited efficacy in ASEAN especially Thailand. This study was aim to evaluate efficacy of high dose PPI Bismuth-containing quadruple therapy with probiotics supplement for *H. pylori* eradication.

**Methods:**

In this double-blind randomized placebo-controlled study, *H. pylori* infected patients were randomized to receive 7-or 14-day high dose PPI- bismuth-containing quadruple therapy with or without probiotics supplement. Probiotic was 37.5 mg Lactobacillus reuteri (Biogaia^®^) in tablet twice daily. *CYP2C19* genotyping and antibiotic susceptibility tests were also done. *H. pylori *eradication was defined as a negative 13C-urea breath test at least 4 weeks after treatment.

**Results:**

100 subjects were enrolled (72 females, 28 males, mean age=54 years). Antibiotic resistance was 15.6% for clarithromycin, 34.1% for metronidazole. *CYP2C19* genotyping was performed in both group and revealed 13%, 50% and 37% for poor, intermediate and rapid metabolizers, respectively. Overall eradication rates of 7-day and 14-day regimens with probiotic were 68% and 96%; P value=0.027. The eradication rate for all patients with poor and rapid metabolizers were 100% with 14-day regimen. 14-day regimen with probiotics can provide 100% eradication with clarithromycin resistance, metronidazole resistance or dual clarithromycin and metronidazole resistance group. Furthermore, the incidence of nausea and vomiting, abdominal discomfort, and bitter taste were significantly lower in patients with probiotics group compared with placebo (6%vs.26%, P=0.002,OR=0.126,95% CI=0.03-0.53; 4%vs.18.0%, P=0.017, OR= 0.155,95% CI=0.03-0.81 and 4%vs.26%, P= 0.001,OR= 0.08, 95%CI= 0.016-0.41, respectively).

**Conclusions:**

The 14-day high dose PPI- bismuth-containing quadruple therapy with probiotic can provide an excellent cure rate for H. pylori infection as first line treatment irrespective of *CYP2C19* and antibiotic resistance pattern. Adding probiotic also significantly reduced treatment-related adverse events and improve the patients’ compliance.

## Introduction

*Helicobacter pylori* (*H. pylori*) is a well-known and proved organism which causes gastric inflammation, peptic ulcer disease (PUD), mucosa-associated lymphoid tissue (MALT) lymphoma and gastric cancer (Rauws and Tytgat, 1990; Bayerdorffer et al., 1995; Parasonet et al., 1991; van der Hulst et al., 1996; Aumpan et al., 2019). Thailand consensus for *H. pylori* management in 2015 suggested that standard triple therapy regimen had cure rate only 80% or less (Mahachai et al., 2016). The alternative first line treatment for* H. pylori* eradication in Thailand is 10-day sequential therapy or 10-day concomitant therapy. However, sequential regimen had complicated daily drug administration and concomitant regimen also associated with taking multiple medication tablets which all were consistent with poor patient compliance and increased adverse drug reactions. Thailand consensus also suggested that adding probiotics in regimen could improve patient adherence and might increase efficacy of eradication rate (Mahachai et al., 2016).

Bismuth-containing quadruple therapy has been recommended as first line treatment in European countries and the United States of America. However, there has limited of evidence about the cure rate for *H. pylori *eradication in ASEAN (Mahachai et al., 2018). This study aim to investigate the cure rate of *H. pylori* eradication treatment with 7-day and 14-day Bismuth-containing quadruple therapy with probiotics (Biogaia Gastrus) for *H. pylori* eradication in Thai patients with non-ulcer dyspepsia. The results will be benefit for *H. pylori* treatment in whole ASEAN countries. *CYP2C19* genotypes have an effect on pharmacokinetics and pharmacodynamics of proton pump inhibitor (PPI) which involved in changing gastric pH and effectiveness of *H. pylori *eradication. Majority of *CYP2C19* genotype in ASEAN patients was related to rapid and intermediate metabolizer (Srinarong et al., 2014). This study also conducted CYP2C19 genetic polymorphisms which could influence treatment outcome.

*Lactobacillus reuteri *(*L. reuteri*) is a gram-positive organism in Lactobacilli group which can change gastric environment from produce lactic acid and 3-hydroxypropionaldehyde (reuterin) which have antibiotic property against *H. pylori* infection (Gerrits et al., 2006). Furthermore, prior studies of *L. reuteri *suggested that this organism could reduce side-effects of *H. pylori* treatment regimen significantly such as abdominal pain, bloating and diarrhea (Dore et al., 2014; Dore et al., 2015; Francavilla et al., 2008). 

## Materials and Methods

*Patients*

Patients age more than 18 years who underwent upper GI endoscopy for evaluation of chronic dyspepsia were included. Patients with non-ulcer dyspepsia, defined as normal upper GI endoscopy or only mild gastritis, were included in the study. Those with a history of prior *H. pylori* eradication, currently receiving PPI, H2-blocker, bismuth group or any kinds of antibiotics within 4 weeks before this study, receiving anticoagulants or NSAIDs, and other serious underlying diseases (eg. heart diseases, major illness, or cancers) were excluded. All participants were written informed consent including understanding and accepting objectives, protocols and treatment complications. This study is already approved by Thammasat University Hospital Ethic committee and was registered to the national clinical registry TCTR20190127003.

*The diagnosis of H. pylori infection*

4 biopsies from antrum of stomach were done during upper GI endoscopy for rapid urease test, *H. pylori *culture, histological examination, *CYP2C19* genotype, Epsilometer test (E-test) or GenoType^®^ HelicoDR. The positive *H. pylori* infection was defined as: positive *H. pylori *culture, or two positive tests (rapid urease test and histology). The *CYP2C19 *genotyping were demonstrated as: rapid metabolizer (RM), intermediate metabolizer (IM), or poor metabolizer (PM). 

*Therapeutic regimens*

All patients were randomized into 4 groups by using a computer-generated list: (1) 7-day high dose PPI-bismuth-containing quadruple therapy with probiotics, (2) 7-day high dose PPI- bismuth-containing quadruple therapy with placebo, (3) 14-day high dose PPI-bismuth-containing quadruple therapy with probiotics, or (4) 14-day high dose PPI- bismuth-containing quadruple therapy with placebo. High dose PPI- bismuth-containing quadruple therapy consisted of bismuth subsalicylate 1,048 mg orally twice daily after meal, metronidazole 400 mg orally 3-time a day after meal, tetracycline 500 mg orally 4-time a day after meal and dexlansoprazole 60 mg twice daily before meal. Probiotic was* Lactobacillus reuteri gastrus* (*L. reuteri DSM17938* and *L. reuteri ATCC PTA6475*) in tablet (Biogaia gastrus^®^) dosed 37.5 mg twice daily after a meal, whereas placebo was exactly identical tablet without probiotics. 

*Post-therapy follow-up*

13C-UBT was applied to assess H. pylori eradication in all individual after treatment for at least 4 weeks. Successful of treatment was defined as negative 13C-UBT. Pill count was done, and drug consumption greater than 90% was defined as well adherent. Personal interview with open-ended questions by questionnaire were used to assess adverse events. The likelihood side-effects listed in questionnaires were nausea, vomiting, skin rashes, bitter taste, abdominal discomfort, diarrhea, headache and palpitation. Therapy-related side effects were defined as new symptoms and worsening of pre-existing symptoms during the treatment period. Side effects severe enough to disrupt patients’ activity from normal life and require hospitalizing were defined as serious events. 

**Figure 1:**
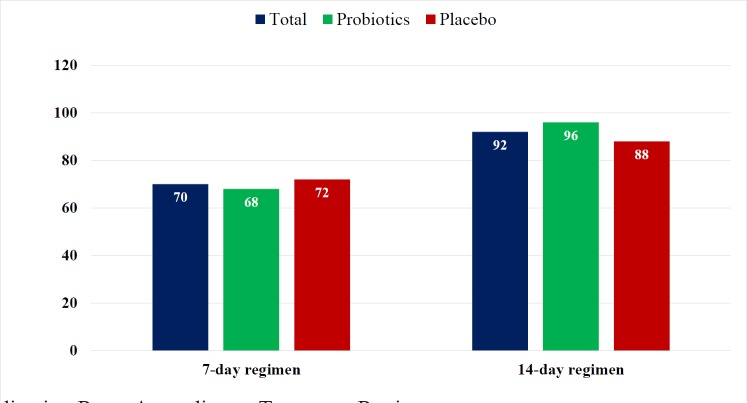
The Eradication Rates According to Treatment Regimens

**Figure 2:**
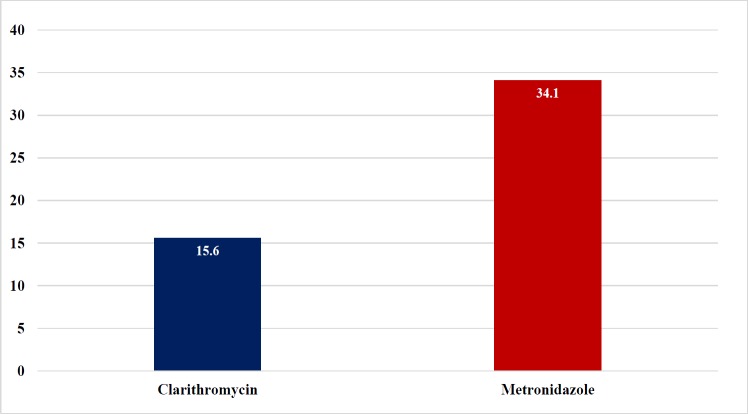
The Prevalence (%) of Antibiotics Resistance Determined by E-test and Genotype HelicoDR

**Figure 3:**
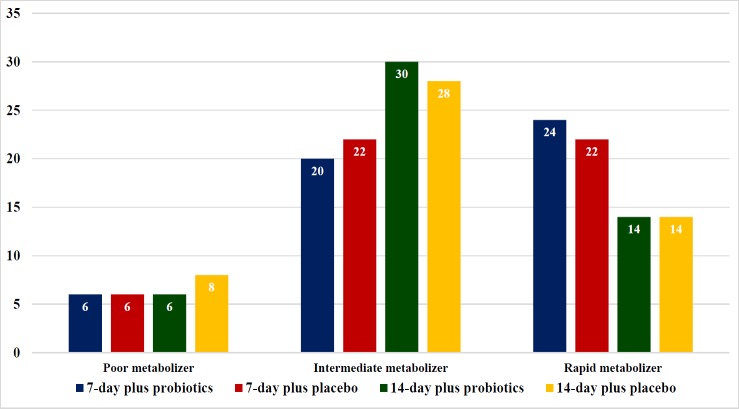
The Prevalence (%) of *CYP2C19* Genotype

**Table 1:** Baseline Demographic Data of All patients

Characteristic data	7-day regimen N = 50	14-day regimen N = 50	P value
Age (years)	56	52	-
Male (%)	17 (34%)	11 (22%)	0.181
Underlying disease			
Diabetes	2 (4%)	4 (8%)	0.40
Dyslipidemia	3 (6%)	9 (18%)	0.065
Hypertension	5 (10%)	12 (24%)	0.062
Smoking	2 (4%)	0 (0%)	0.153
Alcohol consumption	4 (8%)	4 (8%)	1.0

**Table 2:** CYP2C19 Genotype and Eradication Rates

CYP2C19 genotype (N=100)	7-day plus probiotics (N=25)	7-day plus placebo (N=25)	14-day plus probiotics (N=25)	14-day plus placebo (N=25)
PM (N=13; 13%)	3 (66.67%)	3 (100%)	3 (100%)	4 (100%)
IM No. (N=50; 50%)	10 (90%)	11 (81.81%)	15 (93.33%)	14 (78.57%)
RM No. (N=37; 37%)	12 (58.33%)	11 (72.72%)	7 (100%)	7 (100%)

*number in parentheses are eradication rates

**Table 3:** Antibiotic Resistance Pattern and Eradication Rates

Antibiotic resistance	7-day plus probiotics	7-day plus placebo	14-day plus probiotics	14-day plus placebo
CH resistance (N=12)	2/6 (66.67%)	2/3 (33.33%)	0/1 (100%)	0/2 (100%)
MZ resistance (N=14)	0/2 (100%)	2/4 (50%)	0/4 (100%)	1/4 (75%)
CH and MZ resistance (N=4)	-	0/1 (100%)	0/1 (100%)	0/2 (100%)

*, number in parentheses are eradication rates

**Table 4:** Adverse Events Comparing between Probiotics and Without Probiotics Regimens

Adverse event	7-day regimen + L. reuteri	7 days regimen Placebo	P value	14-day regimen + L.reuteri	14-day regimen + placebo	P value
Nausea Vomiting	12%	12%	1.0	6%	26%	0.002
Abdominal discomfort	4%	10%	0.221	4%	18%	0.017
Diarrhea	0	4%	0.149	0	0	-
Fatique	6%	2%	0.297	6%	8%	0.684
Dizziness	6%	6%	1.0	8%	8%	1
Bitter taste	14%	12%	0.747	4%	26%	0.001

*Statistical analysis*

The eradication rate of treatment regimen was estimated to be more than 90%. Treatment success was defined as a cure rate more than 95% (grade A) as described before (Graham et al., 2007), and failure as a cure rate of less than 90% per protocol. Chi-squared, Fisher’s exact, and student’s t-test were used to compare the demographic characteristics and frequencies of side-effects where appropriate. Statistic significant defined as P-value less than 0.05. This study was approved by our local ethics committee, and was conducted according to good clinical practice guideline, as well as Declaration of Helsinki. 

## Results

Total of 100 patients were enrolled in this study, there were 72 females and 28 males which mean age of 54 years. The baseline demographic data were shown in table 1.

*Eradication of H. pylori infection*

Both intention-to-treat and per-protocol analyses results were similar because of no dropping out during study period. *H. pylori* eradication rates in 7-day regimen and 14-day regimen with probiotic were 68% and 96% respectively, P value = 0.027 (Figure 1). 

Antibiotic susceptibility tests (Figure 2.) were performed in 68 strains (27 from E-test and 41 from GenoType^®^ HelicoDR), which have been demonstrated in, 15.6% for clarithromycin, and 34.1% for metronidazole. Interestingly, there were no tetracycline and amoxicillin resistance in this cohort. *CYP2C19* genotyping was performed in both group and revealed 13%, 50% and 37% for poor, intermediate and rapid metabolizers, respectively (Figure 3). 14-day regimen can provide eradication rate from 78.57% - 100% in all *CYP2C19* genotypes patients in our study (Table 2). Nevertheless, 14-day regimen with probiotics can provide 100% treatment with clarithromycin resistance, metronidazole resistance or both clarithromycin and metronidazole resistance group respectively (Table 3.)

*Adverse events*

Bitter taste, nausea and vomiting, black stool and abdominal discomfort are common adverse events in these regimens. All adverse events were demonstrated in Table 4. The incidence of nausea vomiting, abdominal discomfort, and bitter taste were significantly lower in patients with probiotics compared to placebo (6% vs. 26%, P=0.002, OR= 0.126, 95% CI= 0.03-0.53; 4% vs. 18.0% P=0.017, OR= 0.155, 95% CI= 0.03-0.81, and 4% vs. 26%, P= 0.001, OR= 0.08, 95%CI= 0.016-0.41, respectively).

## Discussion

Many epidemiological and experimental studies were supported significant association between *H. pylori* infection and chronic gastritis, peptic ulcer disease, and gastric cancer (Vilaichone and Mahachai, 2001; Vilaichone et al., 2006; Ford et al., 2014: Mahachai et al., 2018). Prevalence of *H. pylori *infection is ranging from 20% in Malaysia, 21-54% in Thailand, 31% in Singapore, >50% in Myanmar, Laos and Vietnam (Vilaichone et al., 2018). Gastric cancer is the fourth most common cancer worldwide and more than 70% of cases occurs in East Asia and developing countries. The treatment outcome are also grave because of advanced stage of presentations (Vilaichone et al., 2001; Vilaichone et al., 2006; Ford et al., 2014; Rahman et al., 2014). Nowadays, *H. pylori* eradication with standard triple therapy was reported to be ineffective (<80%) in several countries (Chey et al., 2017; Mahachai et al., 2018, Chotivitayatarakorn et al., 2017) due to rising of antibiotic resistant’s. New effective regimen might be required to improve treatment of this important bacterium.

Bismuth has long been known to have an antibacterial activity and prevent bacterial colonization to gastric epithelial and has no prior resistance reported to *H. pylori. *Bismuth has acceptable side effects such as nausea, vomiting, numbness and metallic taste. Another important side effect is a black stool that should be warning not to be from upper GI bleeding (Vilaichone et al., 2006). Currently, bismuth-containing quadruple regimen is promoted to treat *H. pylori* infection as effective first-line regimen in Europe and United States of America (Malfaertheiner et al., 2017, Chey et al., 2017) but limited data in Asia. Our prior study revealed that quadruple therapy had limited efficacy for *H. pylori* treatment in Thailand due to poor compliance, high rate of metronidazole resistance and *CYP2C19* genotype RM (Vilaichone et al., 2015), However, this study demonstrated high efficacy of 14-day high-dose PPI- bismuth-containing quadruple therapy with probiotic for H. pylori eradication. Adding probiotic might be improve patient’s compliance and high dose PPI could overcome the effect of *CYP2C19* genotype that lead this regimen had more effectiveness and better adherence than standard quadruple therapy.

Probiotics are live microorganism that provide benefit to human health both of digestive tract and immune system. Many studies revealed that probiotics could decrease some adverse effects of the *H. pylori* treatment regimens (Fuller, 1991; Otles et al., 2003). The possible mechanism of probiotics is restoring the equilibrium of intestinal floras previously altered by the combination of antibiotics in treatment regimen (Armuzzi et al., 2001; Srinarong al., 2014).

*L. reuteri* is *Lactobacilli* group which can change gastric environment from lactic acid and 3-hydroxypropionaldehyde (reuterin) production that have antibiotic property against *H. pylori* infection (Dore et al., 2014; Dore et al., 2015). Prior studies of *L. reuteri* suggested that this organism could reduce side-effects of *H. pylori *eradication regimen significantly, for example, abdominal pain, bloating and diarrhea (Dore et al., 2014; Dore et al., 2015; Francavilla et al., 2008). Our study also demonstrated that L. reuteri was not only improve eradication rate but also decreased some side effects during treatment especially nausea and vomiting, abdominal discomfort and bitter taste. By decreasing these adverse effects, adding probiotics might increase compliance by helping patients complete eradication regimen. 

In conclusion, 14-day High dose PPI- bismuth-containing quadruple therapy plus L. reuteri provide high cure rate of *H.pylori* infection in Thai patients with non-ulcer dyspepsia regardless of *CYP2C19* and antibiotic resistance pattern. Adding probiotics also decreased side effects during the treatment.
